# Theta-alpha EEG phase distributions in the frontal area for dissociation of visual and auditory working memory

**DOI:** 10.1038/srep42776

**Published:** 2017-03-07

**Authors:** Masakazu Akiyama, Atsushi Tero, Masahiro Kawasaki, Yasumasa Nishiura, Yoko Yamaguchi

**Affiliations:** 1 Research Institute for Electronics Science, Hokkaido University, Sapporo, Japan; 2 Institute of Math for Industry, Kyushu University, Fukuoka, Japan; 3 Department of Intelligent Interaction Technology, Graduate School of Systems and Information Engineering, University of Tsukuba, Tsukuba, Japan; 4 Rhythm-based Brain Information Processing Unit, RIKEN BSI-TOYOTA, Collaboration Center, Saitama, Japan; 5 WPI Advanced Institute for Materials Research Tohoku University, Sendai, Japan; 6 Neuroinformatics Japan Center, RIKEN Brain Science Institute, Saitama, Japan

## Abstract

Working memory (WM) is known to be associated with synchronization of the theta and alpha bands observed in electroencephalograms (EEGs). Although frontal-posterior global theta synchronization appears in modality-specific WM, local theta synchronization in frontal regions has been found in modality-independent WM. How frontal theta oscillations separately synchronize with task-relevant sensory brain areas remains an open question. Here, we focused on theta-alpha phase relationships in frontal areas using EEG, and then verified their functional roles with mathematical models. EEG data showed that the relationship between theta (6 Hz) and alpha (12 Hz) phases in the frontal areas was about 1:2 during both auditory and visual WM, and that the phase distributions between auditory and visual WM were different. Next, we used the differences in phase distributions to construct FitzHugh-Nagumo type mathematical models. The results replicated the modality-specific branching by orthogonally of the trigonometric functions for theta and alpha oscillations. Furthermore, mathematical and experimental results were consistent with regards to the phase relationships and amplitudes observed in frontal and sensory areas. These results indicate the important role that different phase distributions of theta and alpha oscillations have in modality-specific dissociation in the brain.

Brain oscillations are closely associated with a range of cognitive tasks. Working memory (WM) is involved in simple short-term memory retention, such as in needed in delayed sample-matching tasks, as well as in mental manipulation, such as mental arithmetic or mental rotation. Increased theta and alpha amplitudes in the frontal area is common to all these tasks^1^,^2^,^3^,^4^,^5^,^6^,^7^,^8^,^9^,^10^,^11^.

In an earlier study, we examined electroencephalographic (EEG) data from participants who engaged in auditory and visual working memory tasks (WMTs). We confirmed task-related increases of alpha and theta amplitudes in the frontal area, and also found that amplitudes increased in the temporal, parietal, and occipital areas. Interestingly, coupling between frontal and temporal areas only occurred during the auditory WMT, whereas it was evident between frontal and parietal areas only during the visual WMT. This finding was partially consistent with other reports in which task switching activity was observed in the prefrontal area^12^,^13^,^14^. However, although the prefrontal area exhibited similar behavior during both types of memory task, temporal and parietal areas displayed modality-specific coupling. The mechanisms for this modality-specific coupling remain unclear. In the current study, we first report that phase distributions in frontal alpha and theta oscillations differ depending on the memory task. Then, we mathematically and experimentally demonstrate that these phase differences provide the key to understanding why modality-selective coupling of alpha and theta activity occurs in these tasks.

## Material and Methods

In the current study, we reanalyzed EEG data from our previous publication^10^.

### Original experimental procedures

**Figure 1:**
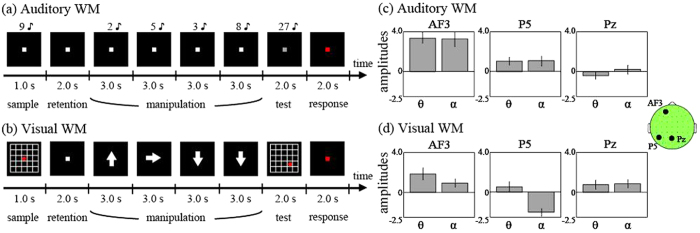
Task procedures of (**a**) auditory and (**b**) visual WM conditions. Subject-averaged theta (4–6 Hz) and alpha (10–12 Hz) amplitudes at frontal (AF3), temporal (P5), and parietal (Pz) electrodes under (**c**) auditory and (**d**) visual WM conditions.

Fourteen healthy right-handed volunteers (10 males and 4 women, 27.9 ± 6.8 years) took part in a WM task with auditory and visual conditions (24 trials each). The RIKEN Wako Institute institutional review board approved the research plan submitted by the authors (MK and YY) for use of human data in experiments designed to measure behavior and EEG. The study was approved by the RIKEN Ethical Committee (in accordance with the Declaration of Helsinki) and all participants gave written informed consent before the experiments. Under the auditory WM condition, participants engaged in mental addition of sequential, aurally presented single-digit numbers (Fig. 1a). Under the visual WM condition, participants memorized the location of a red circle shown in a 5 × 5-square grid on a computer screen; they then moved the red circle in their minds according to the direction specified by a white arrow that appeared onscreen (Fig. 1b). Both conditions included a 1-s stimulus presentation period and a 2-s mental manipulation period in which the stimulus was not present (stimulus-hide). The auditory and visual manipulations were repeated 4 times in each trial. We recorded 62-ch EEG data during the WM tasks, using Ag/AgCl electrodes of an electrode cap (EasyCap, Brain Product, Germany). The sampling rate was 500 Hz. Reference electrodes were placed on the right and left earlobes. Two electrodes were used for recording electrooculographs (EOG), each placed 1 cm from opposite eyes. Data were amplified with a Neuroscan^TM^ SynAmps 2 amplifier (Neuroscan EI Paso, TX, USA) and bandpass-filtered (0.1–50 Hz).

For preprocessing of EEG data, we used infomax independent component analysis (ICA) and removed the ICA components that were significantly correlated with EOG data (i.e., eye-movement and eye-blink artifacts). Moreover, we performed current source-density analysis at each electrode and spherical Laplace analysis to the voltage distribution on the scalp surface (order of the spline: *m* de; maximum degree of the Legendre polynomial: *n* axim; precision: 10^−5^) to determine the volume conduction, which was then also removed from the EEG data.

Time-frequency amplitudes and phases were calculated by wavelet transforms using Morleteq functions (number of cycles: 7; range; 1–20 Hz with 0.5-Hz steps) after removing the artifacts and volume conduction.

### Current experimental procedures

In the current study, we examined the detailed relationships between theta and alpha phases in the frontal areas, as well as in the temporal (for the auditory WM) and parietal (for the visual WM) areas. Results in Kawasaki, *et al*.^10^ had shown that, compared with the baseline 1-s intertrial intervals (ITIs), average theta for first one second of the stimulus-hide period were higher in the frontal (peak electrode, AF3; z = 4.26, *p* < 0.01; correction of Bonferroni multiple comparison) and temporal (peak electrode, P5; z = 2.03, *p* < 0.05; non correction) areas during the auditory condition (Fig. 1c) and in the frontal (peak electrode, AF3; z = 3.53, *p* < 0.01; correction of Bonferroni multiple comparison) and parietal (peak electrode, Pz; z = 2.04, *p* < 0.05; non correction) areas during the visual condition (Fig. 1d). Thus, we selected the peak electrodes—AF3, P5, and Pz—as representative of frontal, temporal, and parietal areas, respectively. We selected 6 Hz and 12 Hz as the theta and alpha frequencies because Kawasaki, *et al*.^10^ showed that within the theta and alpha bands, these frequencies exhibited the highest respectively amplitudes. At the same time points in all manipulation periods, we calculated the probability that that each phase (6-Hz or 12-Hz) would occur, ranging from −π to π (with π/12 steps). Similarly, we calculated the probabilities of their occurrence during the baseline ITI.

To examine the differences in theta-alpha phase relationships between the auditory and visual WM, we used the phase bifurcation index which reflects the differences in phase distribution between different conditions (i.e. auditory WM and visual WM in this study)^15^. If the phase bifurcation index is high, the phase distribution is thought to be locked each other and different between conditions. The results showed that the phase bifurcation indices for the auditory WM and visual WM manipulation periods were significantly higher than those for the ITI periods at the frontal electrode (z = 2.41, *p* < 0.05; correction of Bonferroni multiple comparison). Although the statistical values were not significant, the phase bifurcation indices during the manipulation periods at the parietal and temporal electrodes were higher than those for the ITI periods (P5: z = 2.05, p = 0.05; Pz: z = 1.82, p = 0.06; non correction). These results suggested that the different theta-alpha phase relationships between conditions was not due to noise.

The data showed significant enhancements of theta-phase synchronization between frontal and parietal areas during the visual WM (z = 3.65, p < 0.01; correction of Bonferroni multiple comparison) but not the auditory WM (z = 1.60, p = 0.06). In contrast, theta-phase synchronization between frontal and temporal areas significantly increased during the auditory WM (z = 2.65, p < 0.05; correction of Bonferroni multiple comparison) but not the visual WM (z = 0.72, p = 0.24). The alpha-phase synchronizations were not significant during either the visual WM (frontal-parietal, z = 1.42, p = 0.08; frontal-temporal, z = 1.43, p = 0.08) or auditory WM (frontal-parietal, z = 0.56, p = 0.26; frontal-temporal, z = 1.55, p = 0.07).

**Figure 2:**
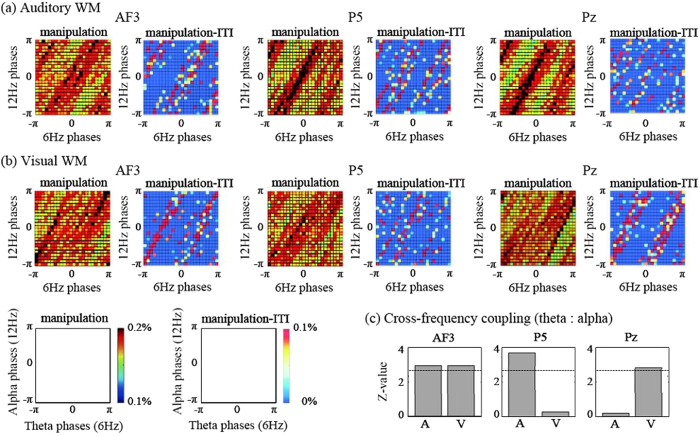
Cross-histograms of probability distributions between the theta (6 Hz) and alpha (12 Hz) phases for the manipulation periods and for differences between the manipulation periods and ITI at AF3, P5, and Pz electrodes under (**a**) auditory and (**b**) visual WM conditions. (**c**) *Z* values of the CFCs between the theta and alpha phases at AF3, P5, and Pz electrodes under auditory (A) and visual (V) WM conditions. The dotted lines denote the threshold values (*P* < 0.01).

For the cross-frequency analyses, the cross-histograms between the theta and alpha phases revealed 1:2 relationships at many electrodes (Fig. 2a,b). In particular, compared with the phases for the baseline ITI, those for the manipulation periods strongly converged on 1:2 concentrations at the AF3 and P5 electrodes under auditory WM and at the AF3 and Pz electrodes under visual WM. Interestingly, the 1:2 phase concentrations at the AF3 electrode clearly showed different differences between the 2 conditions; 0 and π/2 during the auditory and visual WM conditions, respectively.

To identify cross-frequency couplings (CFCs)—the statistical relationships between theta and alpha phases—we applied a modified phase-synchronization index formula^16^. If the CFC increases, the different oscillatory activities are thought to be synchronized and to integrate the separate brain functions. Because the theta and alpha phases showed 1:2 relationships, we used Δ*ϕ*_6Hz-12Hz_ (*t, j*) as the phase difference between twice the theta phases (2 × *ϕ*_6Hz_) and the alpha phase (*ϕ*_12Hz_) at the *j*th electrode on each time point *t* and calculated the CFC values using the following equation:


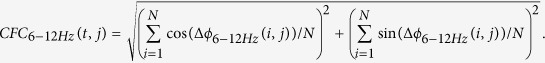


We applied the bootstrap method to the CFCs of each subject’s trials, because of the limitation of the number of sampling data, that is the small number of subjects and trials in this study. Future studies should address the issues. Subsequently, we used the nonparametric Wilcoxon signed-rank test to compare the corrected CFC data obtained during manipulation with the baseline data obtained during the ITI. These analyses were almost identical to those in our previous study^17^. We found that under both the auditory and visual WM conditions, the CFCs at the AF3 electrode were significantly higher during the manipulation periods than during the ITI (Fig. 2c, p < 0.05; correction of Bonferroni multiple comparison). However, at the P5 and Pz electrodes, significant CFCs were only observed under the relevant task conditions. The CFC on the task-relevant electrodes (i.e. P5 for auditory WM and Pz for visual WM conditions) was significantly higher than the CFC on the task-irrelevant electrodes (i.e. P5 for auditory WM and Pz for visual WM conditions), respectively (auditory WM: z = 2.35, p < 0.05; visual WM: z = 2.92, p < 0.01).

Our EEG experiments demonstrate the importance of theta and alpha phase relationships, especially at the frontal areas during WM manipulations of different sensory modalities. The theta and alpha phases were functionally coupled during WM manipulations in the frontal areas, as well as in task-relevant posterior areas, suggesting that interconnectivity of interactions between the executive functions and storage systems^9^,^10^. Notably, the 1:2 theta and alpha phase relationships described here showed shifts between auditory and visual WM conditions, although the theta and alpha amplitudes commonly increased in both of these conditions as reported previously^3^,^7^. Such frontal-phase shifts might lead to modality-specific theta phase synchronizations between the frontal and posterior areas. In order to validate the EEG findings, we next attempted to compute and simulate a mathematical model representing the WM functions.

There are possibilities that the increase in amplitudes would cause phase estimation more accurate and then the stronger theta PSI and theta-alpha CFC. Here, we additionally conducted the correlation analyses between the amplitudes and theta PSI or theta-alpha CFC. As results of PSI, the PSI between frontal and temporal electrodes were not significantly correlated with the frontal theta (r = 0.38; p = 0.17) and temporal theta (r = 0.27; p = 0.35) powers under the auditory WM condition. Moreover, the PSI between frontal and parietal electrodes were not significantly correlated with the frontal theta (r = 0.31; p = 0.28) and parietal theta (r = 0.30; p = 0.27) powers under the visual WM condition. In results of CFC, there were not significant correlation between them in frontal, temporal, and parietal electrodes in visual WM (AF3: r = 0.39, p = 0.17; P5: r = 0.15, p = 0.61; Pz: r = 0.24, p = 0.40) and auditory WM (AF3: r = 0.32, p = 0.26; P5: r = 0.26, p = 0.36; Pz: r = 0.24, p = 0.41) conditions. These results suggested that both the PSI and CFC would not be due to the power enhancements.

This study focused on the 6 Hz-12 Hz CFC, because the CFC showed the highest statistical values in the WM conditions among the other frequency CFC, in comparison to the baseline (see Table 1). Furthermore, this study applied the region of interest analyses and therefore focused on the analyses on the three representative electrodes, because our previous study has shown the highest theta and alpha activities at the electrode in the topographies (Kawasaki, *et al*.^10^). In future study, the whole brain analyses and the other brain measurements with high spatial resolution (e.g. functional magnetic resonance imaging) could provide the detailed mechanisms.

### Mathematical Model

In this section, we outline our mathematical model. The previous section described the relationship between theta and alpha oscillations observed experimentally at the AF3 and P5 or Pz electrodes. Theta and alpha amplitudes increased at AF3 during both tasks, with a different phase distribution occurring during each task. An important question arising from the findings of that experiment was: what mechanism does the brain employ for modality-specific coupling between AF3 and P5 (or Pz)?

**Figure 3:**
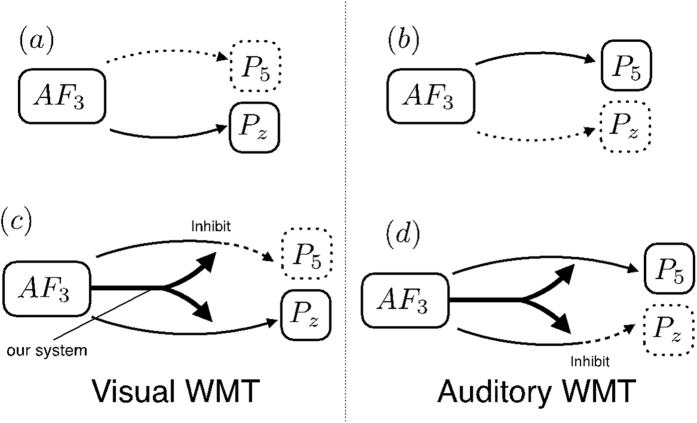
The arrows in the figure on the right indicate the flow of signal transduction; solid arrows indicate the directions of traveling signals, and broken arrows indicate signals that are not traveling. The lines surrounding the lettering (solid or broken) indicate whether or not theta and alpha amplification occurs. (**a**) Global coupling occurs between AF3 and Pz. (**b**) Global coupling occurs between AF3 and P5. (**c**) The phase difference generated in AF3 acts as a signal for selective global coupling of Pz only. (**d**) The phase difference generated in AF3 acts as a signal for selective global coupling of P5 only.

In light of previous reports^12^,^13^,^14^, we proceeded on the assumption that AF3 is the trigger for signal transduction to P5 (or Pz). We also assumed that theta oscillations are responsible for this signal transduction. We have described these assumptions in other recent publications^6^,^10^. We reasoned that a full consideration of the extent to which signals are output by AF3 and transmitted downstream to P5 and Pz was required to answer the question. We first provide a simple summary of our experiments. The right-side arrows in Fig. 3 indicate the flow of signal transduction; solid arrows indicate the directions of traveling signals, and broken arrows indicate signals that are not traveling. The lines surrounding the lettering (solid or broken) indicate whether or not theta or alpha amplification occurs. For example, during the auditory WMT, global coupling occurs between AF3 and P5, as shown in Fig. 3(b). However, if no difference in signals is observed between the two WMTs (corresponding to no difference in phase differences), modality-specific branching as shown in (a) and (b) should not occur. This view is based on the fundamental principle that a single input cannot result in multiple outputs. To resolve this paradox, we propose the mechanism shown in Fig. 3(c).

In this mechanism, the phase difference generated at AF3 acts as a signal for selective global coupling with P5 only. According to this mechanism, although the same signal travels from AF3 to both P5 and Pz, global coupling is caused by the selective inhibition of signal transduction in midstream. The usefulness of our mathematical model depends on its capacity to recreate this mechanism.

**Figure 4:**
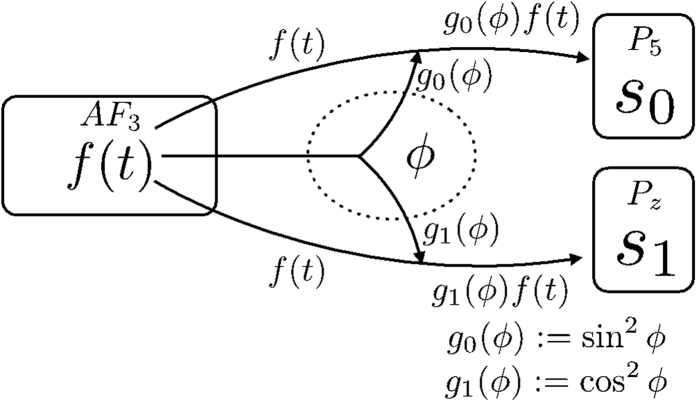
A schematic diagram of the mathematical model. The arrows in the figure indicate the flow of signal transduction.

Posing the problem in this way clarifies the points we sought to demonstrate. A schematic diagram of the mathematical model we finally propose in light of these assumptions is shown below (Fig. 4).

Here, *f(t*), s_0_, and s_1_ are the brain oscillations at AF3, P5, and Pz, respectively.


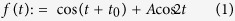



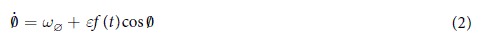



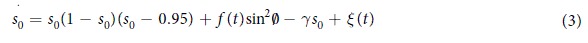



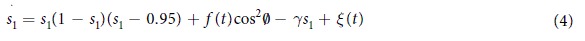


### Equation (1)

Theta and alpha oscillation frequencies have been experimentally shown to be approximately 6 and 12 Hz, respectively. This relationship, in which the ratio between theta- and alpha-oscillation frequency is approximately 1:2, is rendered in a simplified form in Equation 1. Here, the theta oscillation component of the brain oscillation at AF3 is expressed as cos(*t *+* t*_0_) and the alpha oscillation component as *A*cos2*t*. Because both the theta and alpha oscillations were strongly amplified during both WMTs in our experiments, and the duration that this continued was longer (order of seconds) than that of theta- and alpha-phase cycles (order of milliseconds), the amplitude is regarded as constant over time in this equation. *t*_0_ is the phase difference, and as shown in our experiments, it can be replaced by 0 in the auditory WMT and pi/2 in the visual WMT.

### Equation (2)

The signal transduction route has been experimentally shown to open and close depending on phase conditions^18^,^19^,^20^,^21^,^22^. We assume here that the branching pathways exhibit cyclical behavior and that signal transduction to P5 and Pz occurs according to the phase oscillator ϕ. Equation (2) is a differential equation for ϕ derived from this assumption. Based on other experimental results^18^,^19^,^20^,^21^,^22^, ωϕ was set to 1 so as to be at a phase close to that of theta oscillations. Our analysis of this equation showed that ϕ is synchronous with the theta component of *f(t*). This is not inconsistent with the assumption described above that theta oscillations are responsible for the signal. This equation is also known to have robustness even in the presence of a certain amount of external noise. This means that even if other parameters are somewhat varied, as long as the parameter ωϕ is set at a number close to 1, ϕ will be synchronous with the theta component.

### Equations (3) and (4)

We now turn to the derivation of Equations (3) and (4). We have already explained that global coupling between AF3 and P5 occurred during the auditory WMT. This can be regarded as an increase in the amplitude of brain oscillations at AF3 and P5 and a decrease in that of brain oscillations at Pz. We therefore propose a mathematical formula whereby P5 (or Pz) brain oscillations can adopt one of two states: excited or resting. Before describing the formula for P5 (or Pz) in detail, we provide a simple summary of the knowledge required for the deriving this formula.

### Bi-stable Excitability Model

The FitzHugh−Nagumo (FHN) model is a well-known model of neuronal excitability^22^,^23^. The FHN equation is a binary formula expressing the dynamics of neuronal firing and is widely known as a phenomenalistic model of how self-induced excitation can occur under appropriate conditions. This formula was simplified into a two-variable formula by FitzHugh and Nagumo based on mathematical assumptions from the original four-variable formula proposed by Hodgkin and Huxley for reproducing the behavior of membrane potentials in squid axons. It can be expressed as shown below (equations (6) and (7)), in terms of the variables *u* and *v*, indicating excitability and time delay, respectively.


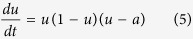



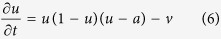



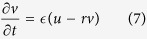


The most characteristic point is the third-order function on the right-hand side of Equation (6). Here, we explain only the first right-hand term. Equation (5) is a bi-stable model with regard to the variable *u* (with 0 < *a* < 1). From this equation, it can be seen that 0, *a*, and 1 are points of equilibrium.

**Figure 5:**
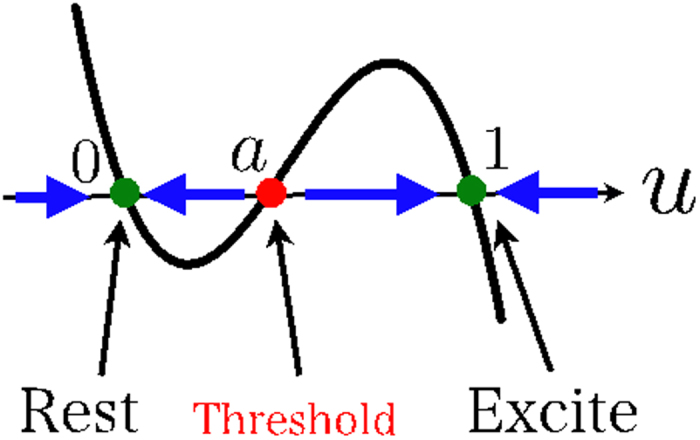
The solid line indicates the function in the equation (5). The green and red dot are stable or unstable point of equilibrium, respectively.

The behavior of *u* is illustrated in Fig. 5; it can be seen that 0 and 1 are stable points of equilibrium, and *a* is an unstable point of equilibrium. Thus, given *u*_0_ is an arbitrary initial value of *u*, if *u*_0_ < *a*, then *u* will be asymptotic at 0, whereas if *a* < *u*_0_ then it will be asymptotic at 1. Given that *u* = 0 is designated as the resting state and *u* = 1 as the excited state, this model is a bi-stable excitability model. The parameter *a* is the excitability threshold, and the closer it is to 1, the more the system tends toward rest, while the closer it is to 0, the more the system tends toward the excited state.

We have used the bi-stable equation described above as the equation for P5 (or Pz). This means that the first right-hand term in equations (3) and (4) becomes a bi-stable excitability model. As a result of this formula, when the brain oscillation *s*_0_ (or s_1_) at P5 (or Pz) is close to 0, the system can be regarded as being in the resting state, and when it is close to 1, it is considered to be in the excited state. When participants are not engaged in a WMT, the amplitude of the brain oscillation at P5 (or Pz) is kept low. In this model, we propose two effects that encourage amplitude damping in the absence of any outside influence on P5 (or Pz). These are:

(i) Setting the threshold *a* at 0.95.

(ii) The addition of a damping term (−γ*u*) as a third right-hand term.

As already described, because the threshold is close to 1, the effect of (i) is that the system tends to transition to the resting state. The effect of (ii) is solely to change the potential aspect with respect to *u* because it brings it closer to the strong stable equilibrium point (*u* = 0). In this simulation, we used both (i) and (ii), so that the system tended to adopt the resting state in the absence of a WMT. In actual experiments, there were large differences between participants, with different times required for P5 (or Pz) to emerge from the excited state. This can be reproduced by varying the two parameters in (i) and (ii) (*a*, γ).

The most important terms in Equations (3) and (4) are the second right-hand terms, representing external input. As described above, the occurrence of modality-specific coupling of P5 (or Pz) means that, at a minimum, we must consider a term with *f*(t) and ϕ for the second right-hand term. The derivation of this term utilizes the prior assumption that theta oscillations are responsible for signal propagation. We considered a range of formula types with theta components or theta-oscillation multiple components from ϕ and found that the phenomenon could be reproduced using the product of *f(t*) and sin^2^ ϕ. The product, rather than the sum, was used because of its property that if one component of a product is zero, the overall value would also be zero. More details are given in the Discussion section.

Because this mathematical model is a model for reproducing an experimental phenomenon, it must also be made robust against noise. We, therefore, added the fourth right-hand term as a white noise term (mean 0, variance 1).

We thus produced a mathematical model from four variables, comprising brain oscillations at AF3, P5, and Pz, and the signal transduction variable ϕ. The experimentally obtained t_0_ was assigned as the control parameter for brain oscillations at AF3, meaning that this model system has only three variables.

### Simulation Results

In this section, we explain the simulation results obtained using our mathematical model. To analyze our mathematical model, we used a highly accurate approximation technique for numerical calculations. Namely, we calculated the next time step value using the 4th order Runge–Kutta method. Parameters other than *t*_0_ were fixed as *A* = 1, ωθ = 1, ε = 0.1, and γ = 0.1, and any changes to these were clearly indicated. Random numbers were assigned as the initial values, and the calculations were repeated. As no changes in qualitative behavior were observed under any circumstances, only the results of the calculations using the initial variables are shown below.

#### Reproducibility of the phenomenon

The phase difference at AF3 observed in the experiments was *t*_0_ = 0 during auditory WMTs and *t*_0_ = π/2 during visual WMTs. We performed simulations for both of these conditions.

**Figure 6:**
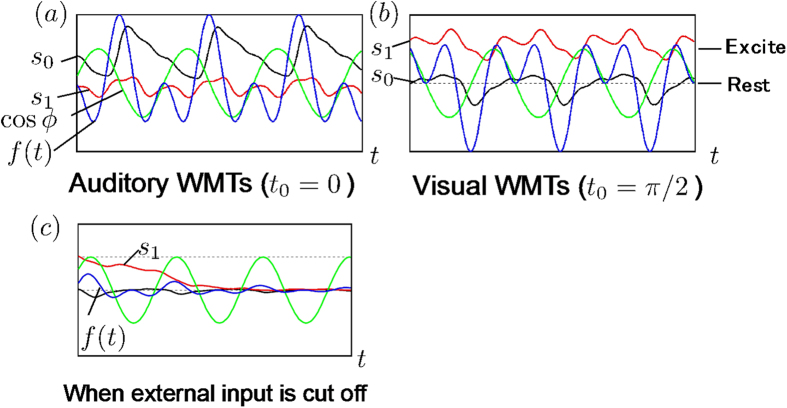
Simulation result of s0, s1, cosϕ and f(t) are shown as solid color black, red, green, and blue lines, respectively. (**a**) This simulation result shows the case of Auditory WMTs. (**b**) This simulation result shows the case of Visual WMTs. (**c**) When external input is absent or cut off.

Figure 6(a) shows the results of the simulation of AF3 brain oscillatory status during the auditory WMT (*t*_0_ = 0). Over time, the variable ϕ synchronized with the brain oscillation at AF3 *f(t*), and the phase difference became locked. Next, when ϕ was made to excite only s_0_ (i.e., the brain oscillation at P5), s_0_ oscillated close to the excited state (s_0_ ≒ 1), and s_1_ oscillated close to the resting state (s_1_ ≒ 0). Our simulation thus reproduced the situation during the auditory WMT.

Similarly, when ϕ was made to excite only s_1_ (the brain oscillation at Pz) to simulate the visual WMT (*t*_0_ = π/2), s_1_ was in the excited state, and s_0_ was in the resting state, as shown in Fig. 6.

Despite its extreme simplicity, our mathematical model is capable of reproducing modality-specific global coupling when a single parameter is varied.

#### When input from AF3 is completely absent or cut off midstream

As stated in the Mathematical Model section, amplitude is known to decrease when external input to P5 (or Pz) is completely absent or cut off partway through. We performed simulations to investigate whether or not this phenomenon could be reproduced in our model. We found that P5 (or Pz) rapidly entered the resting state in either case (see the Fig. 6(c)). Although this was because of the damping factor in Equations (3) and (4), it was also influenced by a noise-dependent effect. Setting the excitability threshold at 0.95 meant that the system tended to enter the resting state. Our mathematical model thus faithfully reproduced the actual phenomenon.

#### Phase difference comparison with experiments

As described in section (1) above, our mathematical model is capable of reproducing the state of modality-specific selective coupling of AF3, P5, and Pz in a manner similar to that seen experimentally. We now describe the results of a more in-depth analysis of these simulation results.

**Figure 7:**
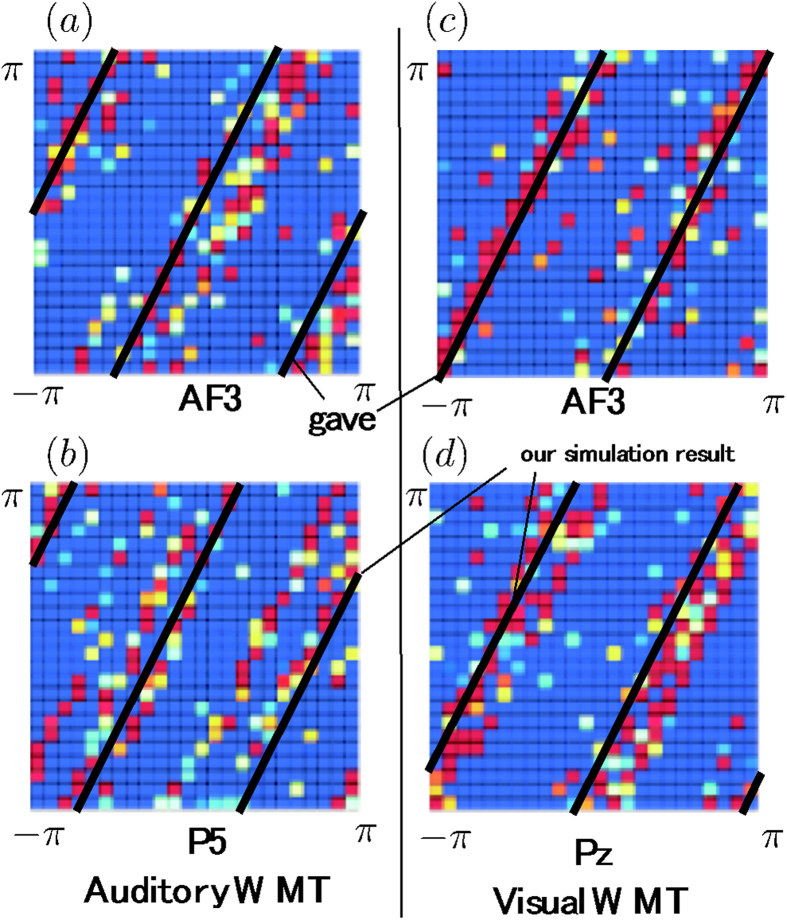
Reproduction of experimental result in mathematical simulations. The solid line shows our simulation result.

The background to Fig. 7(a–d) is a color-coded depiction of the relationships between the theta- and alpha-phase differences during each task, as described in the Experiment section. The phase difference *t*_0_ for the theta and alpha oscillations was almost 0 (Fig. 7(a)) and around π/2 (Fig. 7(c)), respectively. In the simulations, the phase difference *t*_0_ during the auditory WMT was 0. This corresponds to the solid lines in Fig. 7(a). We have previously shown that the system is in the excited state for *s*_0_ (P5) alone and in the resting state for Pz. We conducted a Fourier transformation to investigate which frequency components are included in this P5 waveform (in this simulation, Fourier transformation was used instead of wavelet transformation). This showed that P5 included a multiple component comprised of theta and alpha oscillations. The phase difference for theta and alpha oscillations at P5 is superimposed against the background of the experiment in Fig. 7(b). The solid lines represent the results of the numerical calculations drawn alongside the experimental results, indicating that our mathematical model was capable of reproducing both the selective coupling between AF3 and P5 during the auditory WMT and the phase difference. When we performed a similar verification for the visual WMT, we found almost complete consistency between the phase difference observed experimentally and the results of the mathematical model, as shown in Fig. 7(d). As can be seen from these results, our mathematical model is capable of not only reproducing the phenomenon, as described in (1) above, but also incorporating the phase differences in P5 and Pz brain oscillations.

## Discussion

As demonstrated above, the experimental findings were reproduced using our mathematical model based on the assumption that the phase-difference signal at AF3 was split, with theta oscillations acting as the trigger. In addition to this main result, the model was also capable of incorporating the internal states of P5 and Pz (the relationship between the various phase differences) during both tasks. Here, we consider the derivation of Equations (3) and (4) from a different angle.

### The orthogonal relationship of trigonometric functions

Our Mathematical model is difficult to resolve analyticaly. Our aim is to move the discussion forward by fixing parameters other than *t*_0_ and employing the results of numerical calculations. To consider what sort of term would be most valid as the input term in Equation (3), we consider the temporal mean of input terms. According to our numerical calculations, after sufficient time has passed, ϕ asymptotically becomes *t* + *t*_0_ + pi/2. From this, the size of the input in the second right-hand term in Equation (3) can be calculated as 2π per cycle, according to the following formula.


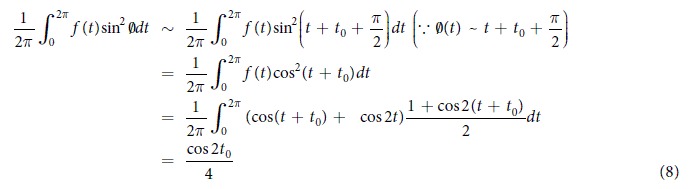


Here, the orthogonal relationship of a trigonometric function has been used in the final transformation of the expression. As can be seen, the temporal mean for the size of the input in the second right-hand term is dependent on *t*_0,_ as cos(2*t*_0_)/4. If this expression for the second right-hand term is multiplied by the theta component, for example by *f(t*)sinϕ, the mean input would be 1/2. This would mean that it was constantly excited irrespective of the phase difference, and would thus be inappropriate because that is not consistent with the phenomenon. When the same consideration is applied to *f(t*)sin^*n*^ϕ, the product with *f(t*) must be a 2*n* multiple component of the theta oscillation (where *n* = 1, 2, …) because the mean input to P5 is dependent on *t*_0_. Taking both of these considerations together, the form of the expression in the second right-hand term should fulfill the following conditions.

- When a WMT is not in progress (i.e., when *f(t*) = 0) the mean size of the input should be zero.
- The mean inputs for ϕ and *f(t*) should include information about the phase difference *t*_0_.
- It should be a type of frequency component that is actually observed in the brain.

Given these conditions, we expressed the form of the input in the simplest terms, as the product of *f(t*) and sin^2^ϕ. As this consideration can also be applied to Equation (4), we propose our final mathematical model in the form of Equations (3) and (4).

### Frequency distributions included in P5 and Pz

**Figure 8:**
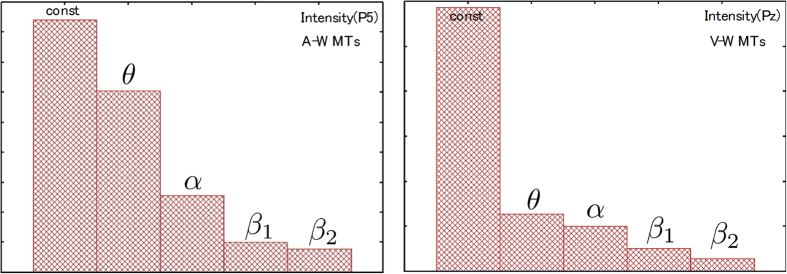
The intensity distributions of simulation results for θ (6 Hz), α (12 Hz), β_1_ (18 Hz) and β_2_ (24 Hz) at P5 and Pz during A-MWTs (left) and V-WMTs (right), respectively.

In part (3) of the Simulations section, we suggested that theta and alpha components are included in P5 (or Pz), and that the relationships of these phase differences are consistent with the experimental evidence. Whether any other components are involved remains as open question. We calculated the intensity values that are a triple component (beta1) and quadruple component (beta2) of the theta oscillations in P5 (or Pz) using Fourier transformation for simulation results. Figure 8 shows the intensity distributions for each frequency at P5 and Pz during A-WMTs and V-WMTs, respectively. The fact that both beta1 and beta2 oscillations were detected is extremely interesting. In the actual experiment, beta1 and beta2 oscillations were observed in a number of participants, but their peaks were low when data for all participants were averaged to obtain data for statistical analysis. The fact that beta1 and beta2 oscillations were detected during simulations also indicates the validity of our mathematical model.

### Robustness to noise

To ensure that our mathematical model reproduced the actual experimental system, the effect of noise was also considered. Detailed parameter adjustment was therefore completely unnecessary for the reproduction of these simulation results. This means that this model possesses similar robustness to that of the actual phenomenon.

## Summary

We experimentally demonstrated modality-specific global coupling between the frontal area (AF3) and the temporal (P5) or parietal (Pz) areas during two WMTs. In this process, consistent phase differences in alpha and theta oscillations were evident in the brain oscillations of the frontal, temporal, and parietal regions. Based on this finding, we assumed that the phase difference in the frontal region was the key to the modality-specific selective coupling, and we produced a mathematical model. Although this mathematical model is an extremely simple three-variable system model, it successfully decoded the frontal phase difference in terms of the oscillator ϕ and reproduced its modality-specific appearance. The relationship between the phase differences included in the temporal and parietal regions was also nearly consistent with the experimental evidence. These results experimentally and theoretically confirm the existing knowledge that brain oscillations in the frontal area induce macrophenomena during a range of cognitive tasks, and they also take this one step further by showing that the phase differences in these brain oscillations act in an essentially important manner^24^.

## Additional Information

**How to cite this article**: Akiyama, M. *et al*. Theta-alpha EEG phase distributions in the frontal area for dissociation of visual and auditory working memory. *Sci. Rep.* **7**, 42776; doi: 10.1038/srep42776 (2017).

**Publisher's note:** Springer Nature remains neutral with regard to jurisdictional claims in published maps and institutional affiliations.

## Figures and Tables

**Table 1:** Statistical p-values about the CFC among theta (4–7 Hz) and alpha (8–12 Hz) phases on Fz, P5, and Pz electrodes between auditory WM (A) or visual WM (V) periods and ITI.

Fz-A	8 Hz	9 Hz	10 Hz	11 Hz	12 Hz	Fz-V	8 Hz	9 Hz	10 Hz	11 Hz	12 Hz
7 Hz	0.424	0.424	0.791	0.057	0.013	7 Hz	0.013	0.791	1.000	0.057	0.002
6 Hz	0.057	0.013	0.057	0.013	0.000^*^^*^	6 Hz	0.424	0.013	0.057	0.424	0.000^*^^*^
5 Hz	0.057	0.013	0.424	0.057	0.057	5 Hz	0.424	0.180	0.180	0.180	0.424
4 Hz	0.002^*^	0.791	0.057	0.424	0.002^*^	4 Hz	0.002^*^	0.791	0.791	0.424	0.424
**P5-A**	**8 Hz**	**9 Hz**	**10 Hz**	**11 Hz**	**12 Hz**	**P5-V**	**8 Hz**	**9 Hz**	**10 Hz**	**11 Hz**	**12 Hz**
7 Hz	0.013	0.424	0.013	0.057	0.180	7 Hz	0.057	0.180	0.057	0.424	0.057
6 Hz	0.013	0.013	0.057	0.057	0.000^*^^*^	6 Hz	0.424	0.057	0.791	0.424	0.791
5 Hz	0.013	1.000	0.013	0.791	1.000	5 Hz	0.424	0.791	0.424	0.180	0.424
4 Hz	0.002^*^	0.791	0.013	0.180	0.002^*^	4 Hz	0.057	0.057	0.180	0.057	0.180
**Pz-A**	**8 Hz**	**9 Hz**	**10 Hz**	**11 Hz**	**12 Hz**	**Pz-V**	**8 Hz**	**9 Hz**	**10 Hz**	**11 Hz**	**12 Hz**
7 Hz	0.424	0.791	0.791	0.057	0.180	7 Hz	0.013	0.057	0.791	0.791	0.013
6 Hz	0.180	0.791	0.013	1.000	0.791	6 Hz	0.013	0.057	0.057	0.013	0.000^*^^*^
5 Hz	0.424	0.791	0.057	0.424	0.057	5 Hz	0.791	0.791	0.057	0.057	0.057
4 Hz	0.002^*^	0.180	0.791	0.180	0.180	4 Hz	0.002^*^	0.013	0.180	0.424	0.180

^**^Showed significantly higher CFC in WM periods than in ITI. ^*^Showed significantly higher CFC in ITI than in WM periods.
